# Assessing Pearl Quality Using Reflectance UV-Vis Spectroscopy: Does the Same Donor Produce Consistent Pearl Quality?

**DOI:** 10.3390/md8092517

**Published:** 2010-09-20

**Authors:** Noldy Gustaf F. Mamangkey, Snezana Agatonovic, Paul C. Southgate

**Affiliations:** 1 Pearl Oyster Research Group, School of Marine & Tropical Biology, James Cook University, Townsville, Queensland 4811, Australia; E-Mail: paul.southgate@jcu.edu.au; 2 School of Pharmacy and Applied Science, La Trobe University, Bendigo, Victoria, Australia; E-Mail: S.Kustrin@latrobe.edu.au

**Keywords:** pearl, pearl quality, pearl oyster, UV-Vis spectroscopy

## Abstract

Two groups of commercial quality (“acceptable”) pearls produced using two donors, and a group of “acceptable” pearls from other donors were analyzed using reflectance UV-Vis spectrophotometry. Three pearls with different colors produced by the same donor showed different absorption spectra. Cream and gold colored pearls showed a wide absorption from 320 to about 460 nm, while there was just slight reflectance around 400 nm by the white pearl with a pink overtone. Cream and gold pearls reached a reflectance peak at 560 to 590 nm, while the white pearl with pink overtone showed slightly wider absorption in this region. Both cream and gold pearls showed an absorption peak after the reflectance peak, at about 700 nm for the cream pearl and 750 nm for the gold pearl. Two other pearls produced by the same donor (white with cream overtone and cream with various overtones) showed similar spectra, which differed in their intensity. One of these pearls had very high lustre and its spectrum showed a much higher percentage reflectance than the second pearl with inferior lustre. This result may indicate that reflectance is a useful quantitative indicator of pearl lustre. The spectra of two white pearls resulting from different donors with the same color nacre (silver) showed a reflectance at 260 nm, followed by absorption at 280 nm and another reflectance peak at 340 nm. After this peak the spectra for these pearls remained flat until a slight absorption peak around 700 nm. Throughout the visible region, all white pearls used in this study showed similar reflectance spectra although there were differences in reflectance intensity. Unlike the spectral results from white pearls, the results from yellow and gold pearls varied according to color saturation of the pearl. The results of this study show that similarities between absorption and reflectance spectra of cultured pearls resulting from the same saibo donor are negligible and could not be detected with UV-Vis spectrophotometry. Nevertheless, this technique could have a role to play in developing less subjective methods of assessing pearl quality and in further studies of the relationships between pearl quality and that of the donor and recipient oysters.

## 1. Introduction

The process of cultured marine pearl production requires a piece of nacre-secreting mantle tissue from a donor oyster (known as saibo), to be grafted with a round nucleus into the gonad of a recipient pearl oyster. The grafted mantle tissue proliferates to form a layer of secretory tissue, which deposits successive layers of nacre onto the nucleus. This process results in the formation of a cultured pearl after approximately two years [1]. Pearls resemble the inner part of the nacreous molluscan shell [2]. The quality of nacre composing the pearl is generally assumed to resemble that of the donor oyster, and donor oysters are chosen primarily on the basis of their nacre quality [3]. Donor selection is assumed to contribute significantly to resulting pearl quality [4]. However, no prior study has compared the quality of pearls resulting from the same donor.

Pearl quality is determined by five primary factors: lustre, color, shape, surface contour and size [2,5–7]. Many of these characteristics are subjective and may depend on individual perceptions [5]. Pearl grading is also labor intensive and requires skill and experience. A pearl grader has to be able to quantify and collate all visual observations and allocate each pearl to a defined grading level. However, techniques are available that may assist in reducing subjectivity in assessing some aspects of pearl quality. For example, UV-visible (UV-Vis) spectrophotometry utilizing a diffuse reflectance accessory can be used to assess material properties based on reflected light. This technique has been extensively applied in a wide-range of chemical analyses of gaseous, liquid and solid materials [8–12]. As UV-Vis light passes through, or is reflected by, a material, specific groups of wavelengths are absorbed and the remaining light is reflected and interpreted by the eye as color. Based on the wavelengths detected within the ultraviolet and eye-visible regions, UV-Vis spectroscopy can rapidly identify chemical properties and color pigments in a non-destructive way. Because pearl color and overtone is determined by the way in which light is reflected through the various outer layers of nacre forming the pearl, this technique has obvious potential application in quantification of pearl color.

UV-Vis spectrophotometry has been used to record the reflectance of pearls from *Pinctada maxima* and *P. margaritifera* [13,14] as well as freshwater pearls [15]. These studies showed that pearl color could be clearly characterized by peaks in reflectance spectra, which are correlated with the presence of particular pigments in the nacre layers [13,14].

This study used UV-Vis spectrophotometry equipped with a diffuse reflectance accessory, to compare pearls produced by *Pinctada maxima*. Emphasis was placed on the application of this procedure in assessing pearls produced by the same mantle donors and pearls of various colors (from white/silver to gold) and overtones.

## 2. Results

**Figure 1:**
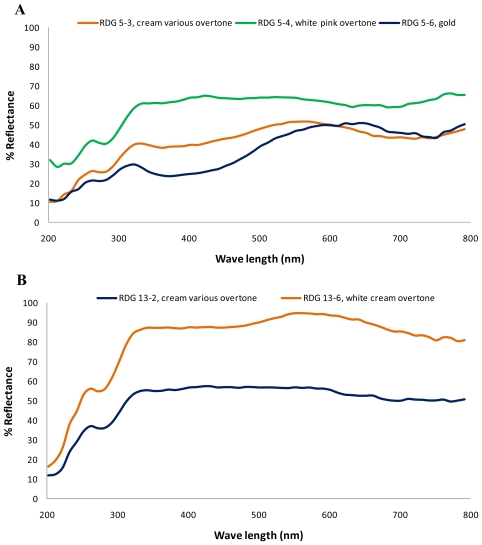
UV-Vis spectral data (reflectance) for (**A**) three *Pinctada maxima* pearls from the same gold donor; and (**B**) two pearls from another gold donor.

Spectral analysis of pearls originating from two donors, represented by the upper and lower graphs in Figure 1, respectively, showed the same reflectance peak recorded in the UV region at 260 nm, followed by absorptions (converse of reflectance peaks) ranging from 270 to 280 nm, then a second peak in the region of 320 to 340 nm. Figure 1A shows variation of spectra of three different pearls from the same gold donor. Cream and gold colored pearls (RDG 5-3 and RDG 5-6) showed a wide absorption from 320 to about 460 nm, while there was just slight reflectance around 400 nm in the white pearl with pink overtone (RDG 5-4). The cream and gold pearls (RDG 5-3 and RDG 5-6) reached a reflectance peak at 560 to 650 nm, while the white pearl with pink overtone had a slightly wider absorption in this area (RDG 5-4). Both cream and gold pearls showed an absorption peak after the reflectance peak: at about 700 nm for the cream pearl and 750 nm for the gold pearl. Figure 1B shows absorption spectra of two pearls resulting from another gold donor. These spectra are similar, but differ in their intensity and in the broad reflectance maximum at around 560 nm for the white pearl with cream overtones (RDG 13-6).

**Figure 2:**
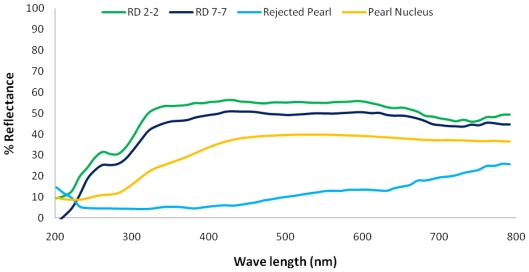
UV-Vis spectral data (reflectance) of two white pearls from different silver donors, a rejected pearl from *Pinctada maxima*, and a pearl nucleus.

The spectra of two white pearls resulting from different silver nacre donors (RD 2-2 and RD 7-7), a rejected pearl (mostly covered with organic material), and a pearl nucleus are shown in Figure 2. The spectra of the two white pearls showed a reflectance at 260 nm, followed by absorption at 280 nm and another reflectance peak at 340 nm. After this peak the spectra for these pearls remained flat until a slight absorption peak around 700 nm. Spectral data recorded for the “rejected pearl” showed that reflectance was not obvious in the UV area (<380 nm; Figure 2). After sloping down to 330 nm, the spectral line rose steadily, passing through the visible region until reaching a maximum at 780 nm. It showed a wide range of absorption. The spectrum of the pearl nucleus showed a slight reflectance peak at 260 nm with a slight absorption in the UV region (Figure 2). The spectral line for the pearl nucleus increased gradually up to a wavelength of 530 nm then decreased only very slightly passing through the visible region.

## 3. Discussion

UV-Vis spectral analysis of pearls has been shown to have considerable potential in characterizing pearl color. For example, yellow-colored pearls and yellow shell nacre from *P. maxima* showed characteristic maximum absorption between 330 and 385 nm while “heat-treated” yellow pearls from *P. maxima* did not [13], allowing treated pearls to be distinguished from natural pearls of similar color. Similarly, visible spectrophotometry has also been used to identify the presence of chromatophores, which show characteristic absorption at 700 nm and provide natural color to Tahitian black pearls from *P. margaritifera* [14]. Absorption at 700 nm is not shown by *P. margaritifera* pearls that are treated to assume similar dark color [15], which again provides a means to distinguish between naturally colored and treated pearls. UV-Vis absorption spectra can also be used to distinguish between the cultured pearls of *P. margaritifera* and *P. maxima* [15]. This study is the first to compare pearls from *P. maxima* produced using the same donor. It shows similar maximum absorption of yellow and gold pearls at around 330–385 nm to those recorded by Elen [13]. The yellow and gold pearls from the same donor (RDG 5-3 and RDG 5-6) in this study also showed similar absorption spectra. The maximum absorption at 330–385 nm is the peak of the wide absorption of 320–460 nm recorded in yellow and gold pearls in this study. Elen [13] reported an absorption similarity in the area of 320–460 nm between yellow shell nacre and yellow/gold pearls. These absorption wavelengths also characterize the differences between yellow shell nacre and white shell nacre from *P. maxima*. Yellow shell nacre showed high absorption between 330–460 nm, which was not evident in white shell nacre [13]. The result of this study also confirms that there is a wide absorption in the same area (330–460 nm) recorded in yellow/gold pearls but no absorption in white pearls. The area between 330–460 nm characterizes yellow-gold pearls from *P. maxima.*

One similarity shown by all nacre covered pearls analyzed in this study (*i.e.*, except the rejected pearl) was a reflectance peak at 260 nm as well as absorptions from 270 to 280 nm in the UV region. A recent study conducted with diffuse reflectance UV-Vis-NIR showed that absorption in the UV region of freshwater pearls collected from two freshwater pearl mussel species (*Hyriopsis cumingi* and *H. schlegeli*) was similarly at 280 nm [16]. The spectral curves recorded for both the rejected pearl and the pearl nucleus in this study do not comply with these characteristic absorption spectra shown by pearls. There was wide absorption across the whole UV and visible ranges by the rejected pearl but just a small absorption by the pearl nucleus. This may indicate that the nacreous part of pearls is primarily responsible for absorption in the UV region. Based on the amount of absorption shown by the rejected pearl, it is likely that the amount of organic matrix plays an important role in the variation of spectral formation in the UV region. The amount of matrix protein composing the surface of the rejected pearl created the wide absorption in both UV and visible regions. The pronounced absorption recorded in the UV region for the pearls used in this study, although not as wide an area as shown by the rejected pearl, indicates that the amount of organic matrix in the nacre of these pearls is lower than in the rejected pearl. Furthermore, a similar reflectance and absorbance in the UV region recorded for the nucleus, although it is just a little, may indicate a trace of organic matrix.

Throughout the visible region, all white pearls used in this study showed similar reflectance spectra although there were differences in reflectance intensity. Unlike the spectral results from those white pearls, the results from yellow and gold pearls varied according to color saturation of the pearl. A weak absorption in the violet region of the visible part marks the spectra of both RDG 5-3 and RDG 13-6. It is followed with a rather strong reflectance of yellow-green color at around 560 to 590 nm. These spectral characteristics confirm the existence of yellow color in the pearls used in this study. Unlike the yellow pearls, strong absorption in the violet region characterized the gold pearl (RDG 5-6). This was followed with a strong reflectance of yellowish-orange color which is visible to the eye. A similar result was also recorded by Elen [13] when he compared the reflectance of natural-color and heat-treated golden cultured pearls that he assumed were from *P. maxima*. He reported broad absorption in the violet region for both natural and treated pearls with yellow and golden colors but not for the white pearls. The absorptions were, however, lower in treated pearls. The same range of absorption was also recorded for the yellow colored lip area of the inner shell surface of *P. maxima* [13].

The color of pearls may come from either the structure of the nacre or the pigments lying in the nacre [16–18]. In the study conducted by Snow *et al.* [17] with pearls from *Pinctada maxima*, the color variations seen in pearls from *P. maxima* were shown to result not only from pigments but also as a result of their structure. They reported that white and gold colors were influenced by differences in the thickness of the edge-band (matrix between the aragonite platelets that make up nacre). They stated that white pearls had a 70 nm edge-band thickness while the edge-band of gold pearls can be up to 90 nm. They also reported that color saturation is determined by the uniformity of nacre structure; the more uniform it is then the more saturate the color. This color appearance has also been detected through UV-Vis reflectance in this study. The more saturated pearl color is indicated by a big variation in wavelengths such as that shown by the gold pearl. Other pearls, *i.e.*, yellow pearls or even white pearls, however, showed small variation in the wavelengths.

The results of this study show that similarities between absorption and reflectance spectra of cultured pearls resulting from the same saibo donor are negligible and could not be detected with UV-Vis spectrophotometry. Although such pearls showed the same absorbance at 280 nm in the UV region, this characteristic was also seen in other pearls tested. Furthermore, this study cannot confirm similarities of certain reflectance intensity characteristics in pearls seeded with saibo from the same donor since there were different reflectance wavelengths and intensities among pearls originating from the same saibo donor. However, this is only one part of the equation; further study is needed to compare the structural and elemental composition of donor shell nacre and the nacre of pearls resulting from saibo from that donor, before firmer conclusions can be made.

UV-Vis spectrophotometry has proven to be a useful tool in prior studies which have used this technique to distinguish between naturally colored and treated cultured pearls and to determine absorption spectra characteristics associated with particular pearl colors [13,15]. While the present study could not identify spectra characteristics of pearls resulting from the same saibo origin, the data indicate that UV-Vis spectrophotometry may also have value in quantifying another pearl “virtue”—lustre. Two pearls used in this study originated from the same saibo donor (RDG 13-2 and RDG 13-6) and showed very similar absorption spectra. They differed, however, in their lustre and this is clearly shown in the intensity of reflectance recorded for each pearl. RDG 13-2 was the only pearl in this study with the highest lustre grading and it showed considerably greater reflectance than all other pearls.

This study assessed a non-qualitative method for determining the characteristics of pearl color. UV-Vis spectroscopy is non-destructive and can be used to determine different pearl colors, pearl origin, color enhancements and treatment, and as a quantitative measure of pearl lustre. This study is the first conducted with a record of the origin of the pearls used, as well as the first to compare pearls produced by saibo resulting from the same donor. This technique could become increasingly important as the pearling industry seeks to develop less subjective methods of assessing pearl quality (grading), it may also become a valuable tool in further studies of the relationships between pearl quality and that of the donor and recipient oysters.

## 4. Experimental Section

**Figure 3:**
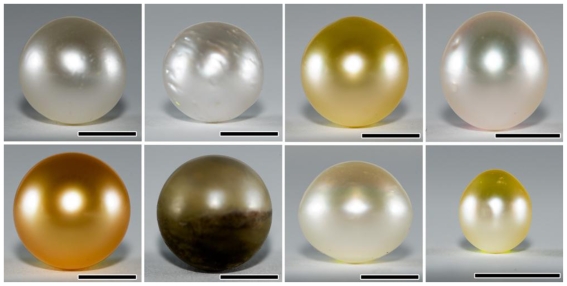
Pearls from *Pinctada maxima* analyzed in this study. **Top** (**left to right**): RD 2-2, RD 7-7, RDG 5-3, and RDG 5-4; **Bottom** (**left to right**): RDG 5-6, RDG 12-6, RDG 13-2, and RDG 13-6. Five pearls were from two donors: RDG 5-3, RDG 5-4 and RDG 5-6 were from the first donor and RDG 13-2 and RDG 13-6 were from the second donor. Scale bars = 5 mm. Pearl nucleus sizes were 5.8–7.0 mm.

Eight pearls from *Pinctada maxima* (seven “acceptable” pearls and one “rejected” pearl) ranging from white to gold in color, together with one white pearl nucleus were used in this study (Figure 3, Table 1). “Acceptable pearls” are pearls appropriate for jewelry purposes while “rejected pearls” are not; they have no commercial value. The rejected pearl used in this study was covered primarily with organic material, not nacre. The studied pearls (Figure 3) were harvested from a commercial pearl farm in Bali, Indonesia and graded using the South Sea Pearl Grading System developed commercially by Atlas Pacific Ltd. (e.g., McGinty *et al.* [19]). Pearl samples with labels RDG 5-3, 5-4 and 5-6 as well as RDG 13-2 and 13-6 were from the same saibo donor while two other pearls were from different donors (RD 2-2, RD 7-7). No treatment was conducted on the pearls to enhance color or lustre after harvest with the exception that they were cleaned with a soft cloth. Nacre thickness was also recorded by subtracting the nucleus diameter from the pearl diameter.

Because of the opaque nature of pearls, spectroscopy measurements were performed using diffuse reflectance techniques: Diffuse Reflectance UV-Vis spectroscopy. The reflectance UV-visible spectra were collected using a Cary 50 UV-Vis spectrophotometer equipped with an external remote Diffuse Reflectance Accessory (DRA) probe (Barrelino™, Harrick Scientific) that can scan an area less than 1.5 mm in diameter. UV-Vis spectra of pearls were acquired in the region 200–800 nm using appropriate baseline correction at approximately 100%. The UV-Vis scan rate was 9600 nm min^−1^. Prior to scanning, the white level was calibrated with Wavelength Reflectance Standard (Labsphere^®^) in which approximately 100% reflectance across the entire spectrum is designated as white reference standard. The spectra were acquired at two different locations on each sample to assess surface homogeneity. To conduct the scanning, each sample was placed onto a stand and scanned with the DRA probe connected to the spectrophotometer. The spectral data were then analyzed and the graphs were smoothed to reduce the noise using the Moving Average method by counting the average of 20 data sets in every fifth wavelength record [20]. The analysis was based mainly on the visible region of the graph (380–750 nm).

## Figures and Tables

**Table 1:** Characteristics of the South Sea pearls produced by *Pinctada maxima* used in this study.

Code	Pearl Color	Pearl Lustre a	Pearl size (mm)	Nacre Thickness (mm)	Donor Color b	Recipient Color c
RD2-2	White	3	10.5	3.2	White	White
RD7-7	White	3	9.5	2.8	White	Yellow
RDG5-3	Cream, various overtone	2	10	3.6	Yellow	Yellow
RDG5-4	White, pink overtone	2	8.5	2.7	Yellow	Yellow
RDG5-6	Gold	2	10.5	4.4	Yellow	Yellow
RDG12-6	Rejected pearl (brown to dark brown)	-	-	-	White	Yellow
RDG13-2	White with cream overtone	3	10.5	3.5	Yellow	Yellow
RDG13-6	Cream, various overtone	1	5	Keshi d	Yellow	Yellow

a Pearl lustre grading factor: 1 = mirror reflection lustre; 2 = somewhat mirror reflection; 3 = chalky appearance.

b Yellow consists of yellow to gold, and white as white or silver.

c Yellow consists of yellow to gold, and white as white or silver.

d A cultured pearl without nucleus, mostly nacreous.
